# Rapid Point-of-care Testing to Inform Intrapartum Treatment of Group B *Streptococcus*–Colonized Women in Uganda

**DOI:** 10.1093/ofid/ofae605

**Published:** 2025-03-10

**Authors:** Juliet Nsimire Sendagala, Melanie Etti, Rose Azuba, Joseph Peacock, Kirsty Le Doare, PROGRESS Study Author Group, Abdelmajid Djennad, Agnes Nyamaizi, Agnes Ssali, Alexander Amone, Amusa Wamawobe, Annettee Nakimuli, Caitlin Farley, Carol Nanyunja, Christine Najuka, Cleophas Komugisha, Dan R Shelley, Edward A R Portal, Ellie Duckworth, Emilie Karafillakis, Geraldine O’Hara, Godfrey Matovu, Hannah G Davies, Janet Seeley, Joseph Peacock, Juliet Nsimire Sendagala, Katie Cowie, Kirsty Le Doare, Konstantinos Karampatsas, Lauren Hookham, Madeleine Cochet, Margaret Sewegaba, Mary Kyohere, Maxensia Owor, Melanie Etti, Merryn Voysey, Moses Musooko, Musa Sekikubo, Owen B Spiller, Patience Atuhaire, Paul T Heath, Philippa Musoke, Phiona Nalubega, Pooja Ravji, Richard Katungye, Ritah Namugumya, Rosalin Parks, Rose Azuba, Sam Kipyeko, Simon Beach, Stephen Bentley, Tim Old, Tobius Mutabazi, Valerie Tusubira, Vicki Chalker

**Affiliations:** Medical Research Council/Uganda Virus Research Institute and London School of Hygiene & Tropical Medicine Uganda Research Unit, Entebbe, Uganda; Institute for Infection and Immunity, St George's, University of London, London, UK; Makerere University—Johns Hopkins University (MUJHU) Research Collaboration, Kampala, Uganda; Department of Livestock and Industrial Resources, Makerere University College of Veterinary Medicine, Animal Resources and Biosecurity, Kampala, Uganda; Institute for Infection and Immunity, St George's, University of London, London, UK; Medical Research Council/Uganda Virus Research Institute and London School of Hygiene & Tropical Medicine Uganda Research Unit, Entebbe, Uganda; Institute for Infection and Immunity, St George's, University of London, London, UK; Makerere University—Johns Hopkins University (MUJHU) Research Collaboration, Kampala, Uganda

**Keywords:** antenatal testing, group B streptococcus, loop-mediated isothermal amplification, molecular diagnostic technique, rapid diagnostic test

## Abstract

**Introduction:**

Maternal Group B *Streptococcus* (GBS) rectovaginal colonization is an important risk factor for invasive disease in neonates, yet availability of culture-based methods for detection is limited in low-resource settings. We evaluated the diagnostic performance of the HiberGene (HG) GBS loop-mediated isothermal amplification (LAMP) assay for the rapid detection of GBS in rectal/vaginal swabs collected from women in Uganda. This work forms a part of the PROGRESS GBS study.

**Methods:**

In phase 1, 1294 rectal and vaginal swabs were collected from pregnant women and inoculated in enrichment (Lim) broth, which was then tested using the HG GBS LAMP assay (*sip* gene target) and culture on chromogenic agar. In phase 2, 166 swabs from nonpregnant women were tested directly (without the enrichment step). For samples with discordant results, an additional method of testing against multiplex real-time polymerase chain reaction assay was used.

**Results:**

Overall, the HG GBS LAMP assay detected more GBS-positive samples (31.3%; 452/1445) than culture-based methods (13.3%; 192/1445). Multiplex polymerase chain reaction–tested results were concordant with LAMP results in 96.3% of cases. The sensitivity and specificity of the LAMP assay, after adjusting for the tiebreaker results of discordant samples, were 94.4% (95% confidence interval, 86.2–99.4) and 99.0% (95% confidence interval, 94.3–100), respectively.

**Conclusions:**

The results of this study demonstrate high sensitivity and specificity of the HG GBS LAMP assay for the detection of GBS rectovaginal colonization in our setting. Given its rapid turnaround time, the HG GBS LAMP assay could appropriately be used to screen women for GBS rectovaginal colonization during labor to enable provision of intrapartum antibiotic prophylaxis.

## Teaser

The HiberGene Group B *Streptococcus* loop-mediated isothermal amplification assay demonstrates high sensitivity and specificity for detection of Group B *Streptococcus* rectovaginal colonization with rapid turnaround time, suggesting its use during labor to enable provision of intrapartum antibiotic prophylaxis in low-resource settings.

Group B *Streptococcus* (GBS), also known as *Streptococcus agalactiae*, is one of the leading causes of neonatal sepsis and meningitis globally with an estimated incidence of invasive GBS disease among infants on the continent of Africa of 1.12 cases per 1000 livebirths [1]. Maternal GBS rectovaginal colonization is recognized as the major risk factor for the development of invasive GBS disease in neonates [2]. In many high-income nations, the burden of invasive GBS disease in neonates is reduced by screening pregnant women for GBS rectovaginal colonization between 35 and 37 weeks of pregnancy and administering intrapartum antibiotic prophylaxis (IAP) to women who are found to be colonized [2, 3]. In such settings, enrichment culture of vaginal and rectal swab specimens is considered the gold standard method for the detection of GBS [2, 3].

In low- and middle-income countries (LMICs), such as Uganda, there are a number of challenges that make the provision of such care unfeasible. First, antenatal clinics often provide services to a large number of women daily, limiting the availability of financial and human resources for antenatal screening [4]. Additionally, while the World Health Organization (WHO) recommends at least 8 antenatal visits during pregnancy, many Ugandan women may only attend an antenatal clinic once during their pregnancy because of the cost of travel, other work or family commitments, or living a far distance from the clinic [4, 5]. Second, the microbiology laboratory infrastructure and expertise to perform GBS screening by enrichment culture is limited [6, 7]. Finally, the process of enrichment culture is labor-intensive with a long turnaround time (TAT) of a minimum of 48 hours [8, 9]. Although polymerase chain reaction (PCR) has the advantage of faster turnaround time, stringent quality control procedures are required to avoid false-positive results that may occur because of contamination, as well as specialist laboratory expertise and equipment that may not be available in all hospitals in LMICs [10].

To overcome many of these situational challenges, point-of-care testing for many infectious diseases such as HIV, syphilis, and malaria has been integrated into antenatal care settings globally. If rapid, accurate, and affordable point of care of testing for GBS colonization could be developed to optimize the administration of IAP, this would minimize GBS transmission risk and greatly improve the disease burden in LMICs. In light of this, we evaluated the diagnostic performance of the HiberGene (HG) GBS loop-mediated isothermal amplification (LAMP) assay for the detection of maternal GBS rectovaginal colonization, comparing it to enrichment culture using the World Health Organization criteria for diagnostic test suitability [11].

This paper forms part of a supplement based on the PROGRESS study. The Progressing Group B Streptococcal Vaccines (PROGRESS) study aimed to describe the causes of infectious mortality and morbidity in pregnancy and neonates, as well as the seroepidemiology of GBS infection—the major cause of neonatal sepsis worldwide—in Kampala, Uganda [12].

## MATERIALS AND METHODS

### Study Setting and Recruitment

This was a prospective diagnostic accuracy study evaluating HG's LAMP assay (index test) (HiberGene Diagnostics, Dublin, Ireland) by comparing its diagnostic performance against enrichment culture (reference test), the current gold standard method of detection [2]. This study was undertaken in 2 phases. In phase 1 of the study, pregnant women were recruited as part of the PROGRESS study (NCT04549220) between 6 August 2019 and 9 March 2020. In phase 2 of the study, nonpregnant women were recruited as part of the TIMING study (NCT04059510) between 9 March and 24 March 2021. Participants were recruited at Kawempe National Referral Hospital (KNRH), a tertiary hospital situated in the north of Kampala, Uganda. KNRH is one of the largest government-funded urban hospitals in Uganda with approximately 80–100 infant deliveries daily [12].

All samples collected during phase 1 were tested by both enrichment culture and LAMP at the Medical Research Council/Ugandan Virus Institute and London School of Hygiene and Tropical Medicine (MRC/UVRI & LSHTM) Clinical Diagnostic Laboratories (CDLS) in Entebbe, Uganda. MRC/UVRI & LSHTM CDLS is Good Clinical Laboratory Practice certified and ISO 15189 accredited and participates in the College of American Pathologists external quality assessment scheme for bacteriology. In phase 2, direct LAMP testing of swab samples was undertaken in KNRH by a trained midwife. Samples were labeled only with a unique identifier that linked the sample to the participant record in the online study database.

### Eligibility Criteria and Sampling

In phase 1, eligible participants included women over the age of 18 years and emancipated minors between ages 14 and 17 years of age delivering a live or stillborn baby at KNRH who were able and willing to give informed consent. In phase 2, consented nonpregnant women older than age 18 years receiving gynecology care at KNRH were included. Full eligibility of participants and sampling methods have been previously published [12]. In brief, eligible participants had a separate swab collected at delivery for LAMP testing and one for culture (phase 1) or dual-headed swab in clinic (with one head sent for LAMP testing and one for culture) (phase 2) taken from the vagina and then the rectum by a study midwife for LAMP testing and enrichment culture either at delivery or at an eligible study visit.

### Laboratory Methods

Phase 1: Individual vaginal and rectal swabs were collected in Amies transport medium and transported to MRC/UVRI & LSHTM CDLS at 2–8 °C and analyzed within 48 hours of collection. Swabs were first inoculated in 3 mL Todd Hewitt with colistin and nalidixic acid (Lim) broth, and incubated in 5% carbon dioxide for 24 hours at 37 °C. An aliquot of inoculated broth (150 µL) was transferred to 3 mL elution buffer followed by analysis by the HG LAMP assay (see the following section) and another 10 μL of the broth was streaked on CHROMagar™ StrepB plates. After overnight culture, the plates were read for presumptive GBS colonies, which appear mauve on the plate. Negative plates were incubated for a further 24 hours to assess for any late growth. Any presumptive isolates underwent Lancefield streptococcal grouping (Oxoid) to confirm GBS.

Phase 2: A dual-headed rectovaginal swab was collected and split such that 1 head was eluted in 3 mL of lysis buffer followed by testing on the HG GBS LAMP assay and the second swab was cultured as outlined for phase 1. Samples that gave discordant results in phase 2 only (either LAMP positive and enrichment culture negative or LAMP negative and enrichment culture positive) underwent tiebreaker testing with the Allplex™ Meningitis-B PCR assay (Seegene, Seoul, South Korea) targeting the *cfb* gene.

HG GBS LAMP assay: A total of 80 µL of diluted Lim broth with eluate (phase 1) or eluted sample (phase 2) was added to a 1 mL cryovial with an enzymatic lysis agent and incubated at room temperature for 20 minutes. The cryovial was transferred to heat block at 105 °C for 5 minutes for denaturation, cooled at room temperature for 5 minutes and then 25 μL of the denatured lysate was added to the HG reaction strip, which contained 2 primers: the *sip* gene and an exogenous bacteriophage sequence used as the assay Extraction Control, together with an intercalating dye. Results were displayed on the machine as “Positive,” “Negative,” or “Invalid.” Positive results displayed in real time from 9 minutes. The run took 40 minutes to complete. The limit of detection of GBS is 784 cells/mL and 0.93 copies/μL of plasmid DNA.

Allplex™ meningitis-B PCR assay: The assay was run according to manufacturer's instructions using the denatured lysate from each sample [13].

All assays were run by independent laboratory technicians who were blinded to the results of the other assays. Results were matched by anonymized study ID by one of the authors (K.L.D.). GBS-positive samples and American Type Culture Collection bacterial reference strains were used as quality controls to test the performance of culture media and assay procedures.

### Statistical Analysis

We determined the sample size based on the precision, sensitivity, and specificity of the index test from the previous study by Curry et al. [14], and a pilot prevalence of 28.8% in our target population [14]. Statistical analysis was performed using Stata version 15 (La Jolla, CA, USA). Frequencies and percentages were used to describe the data with 95% confidence intervals. TAT is described as mean ± 1 standard deviation. We compared means and medians by parametric and nonparametric tests as appropriate and calculated sensitivity and specificity using chi-squared test and 2-tailed Fisher exact test where appropriate. We considered any positive test as a positive sample [14]. A *P*-value of <.05 was considered significant.

## RESULTS

### Diagnostic Evaluation of the HG GBS LAMP Assay

#### HG GBS LAMP Assay Evaluation Using Postenrichment Lim Broth Samples

A total of 1294 vaginal and rectal swabs were tested. Among these, 15 swabs (5 vaginal and 10 rectal swabs) gave invalid results with the LAMP assay and were excluded from the final analysis. There were 147 GBS-positive results on enrichment culture and 384 GBS-positive results using LAMP (rectal swab = 169 and vaginal swab = 215) (Table 1). The mean TAT for LAMP was 68 minutes ± 10 minutes, whereas for enrichment culture, the mean TAT was 3914 minutes ± 646 minutes and the minimum TAT was 2880 minutes ± 720 minutes.

**Table 1.** HG GBS LAMP Assay Evaluation Using Postenrichment Lim Broth Samples Compared Against Enrichment Culture (n = 1279)

HG GBS LAMP (Index Test)	Enrichment Culture (Reference Test)		
	*Positive (+)*	*Negative (−)*	*Total*
Positive (+)	145	239	**384**
Negative (−)	2	893	**895**
Total	**147**	**1132**	**1279**

Abbreviation: HG GBS, LAMP, group B *Streptococcus* HiberGene loop-mediated isothermal amplification.

#### HG GBS LAMP Assay Evaluation Using Direct Swab Samples

A total of 166 rectal and vaginal swabs were tested. There were 45 GBS-positive results detected by enrichment culture and 68 GBS-positive samples detected using the HG GBS LAMP assay (34 rectal swabs and 34 vaginal swabs) (Table 2).

**Table 2.** HG GBS LAMP Assay Evaluation Using Direct Swab Samples Compared Against Enrichment Culture (n = 166)

HG GBS LAMP (Index Test)	Enrichment Culture (Reference Test)		
	*Positive (+)*	*Negative (−)*	*Total*
Positive (+)	41	27	**68**
Negative (−)	4	94	**98**
Total	**45**	**121**	**166**

Abbreviation: HG GBS, LAMP, group B *Streptococcus* HiberGene loop-mediated isothermal amplification.

#### Tiebreaker Testing of Discordant Samples

In phase 2 of the study, there were 27 swabs that were enrichment culture negative and LAMP positive. To determine the true GBS status of these samples, they were each retested with the Allplex™ Meningitis-B PCR assay. Of these 27 swabs, 96.3% (26/27) yielded a positive result with the Allplex™ assay and were therefore considered GBS positive. All samples that yielded a positive result with enrichment culture were considered GBS positive (Table 3).

**Table 3.** Adjusted Performance Evaluation of the HG GBS LAMP Assay After Tiebreaker Testing of Discordant Samples (n = 166)

HG GBS LAMP (Index Test)	GBS Colonization Status		
	*GBS-positive (+)*	*GBS-negative (−)*	*Total*
Positive (+)	67	1	**68**
Negative (−)	4	94	**98**
Total	**71**	**95**	**166**

Abbreviation: HG GBS, LAMP, group B *Streptococcus* HiberGene loop-mediated isothermal amplification.

#### Estimates of Diagnostic Accuracy

The sensitivity and specificity of the HG GBS assay were 98.6% and 78.9%, respectively, with postenrichment Lim broth. The initial sensitivity and specificity of the direct swab samples were 91.1% and 77.7%, respectively; however, the results of the tiebreaker testing of discordant samples with the Allplex™ Meningitis-B PCR assay raised the sensitivity and specificity to 94.4% and 99.0%, respectively. Detailed results are shown in Table 4.

**Table 4.** Estimates of Diagnostic Accuracy of the HG GBS LAMP Assay

	HG GBS LAMP (Index Test) Against Enrichment Culture (Reference Test)					
	Postenrichment Lim Broth		Direct Wwab			
			Before Tiebreaker		After Tiebreaker	
	%	95% CI	%	95% CI	%	95% CI
Sensitivity	98.6	95.2–99.8	91.1	78.8–97.5	94.4%	86.2–98.4
Specificity	78.9	76.2–81.3	77.7	69.2–84.8	99.0%	94.3–100
Disease prevalence	11.5	…	27.1	…	27.1	…
Positive predictive value	37.8	35.1–40.1	60.3	51.8–68.2	97.1	82.6–99.6
Negative predictive value	99.8	99.1–99.9	95.9	90.2–98.4	97.9	94.8–99.2
Accuracy	81.2	78.9–83.3	81.3	74.6–86.9	97.7	94.1– 99.4

Abbreviations: CI, confidence interval; HG GBS, LAMP, group B *Streptococcus* HiberGene loop-mediated isothermal amplification.

## DISCUSSION

This is the first study to investigate rapid diagnostic tests in labor for the assessment of GBS rectovaginal colonization in Uganda and the first to indicate acceptable sensitivity and specificity of the HG GBS LAMP assay, compared to culture, regardless of a pre-enrichment step. Our overall sensitivity and specificity outcomes are consistent with those reported by the manufacturer (97.7% and 100%, respectively) and of other platforms that produce nucleic acid amplification tests (NAATs) for the detection of GBS, such as GeneXpert (98.6% and 95.5%) [15], Panther fusion (95.9% and 99.4%), and Aries (96.6% and 96.3%) [16]. Similarly, sensitivity outcomes for the detection of GBS from Lim broth samples with culture as the gold standard also were consistent with the results of those reported from the same platforms. For direct swab testing, similar sensitivity results were reported in a prior study evaluating diagnostic accuracy the HG GBS LAMP assay in Ireland (92.2%) [14], as well as other studies evaluating rapid molecular-based assays, including the GeneOhm IDI-strep B (91.1%) [17] and quantitative PCR (95.5%) [18]. However, high NAAT sensitivity is not universal; lower test sensitivities have been demonstrated for the AccuProbe assay (ribosomal RNA target) with Lim broth samples (86.5%) [17] and for the Gene pert (*cfb* gene target) with the use of direct swabs (62%) [19]. Such observed differences could be due to different gene targets (*sip, cfb* genes), which has been shown by Carrillo-Ávila et al. to cause a variation in the yield of positive results [18]. Our pre-tiebreaker specificity (79.1%) was less than the estimates provided by reported by Buchan (92.4%) [15], Curry et al. (95.6%) [14], and Carrillo-Ávila et al. (99.1%) [18]. We believe this was due to the ability of the HG GBS LAMP assay to detect bacteria that were no longer viable for culture. Similar to other studies, we demonstrated that enrichment culture likely underestimated GBS positivity in our setting, possibly because of the challenge of implementing enrichment culture where long transportation times to the testing laboratory could have caused loss of bacterial viability [6, 14]. To ensure that our assumptions were valid, we performed rapid testing for GBS using 2 different targets: the *cfb* (Allplex™) and *sip* (LAMP) genes. Similar to a study by Carrillo-Ávila et al. that also compared culture to 2 NAATs that targeted the *cfb* and *sip* genes [18], we found high concordance of 95% between Allplex™ Meningitis-B PCR and the HG GBS LAMP assays, suggesting that the culture negative, HG GBS LAMP positive, Allplex™-positive samples were true-positive samples.

Our results are similar to those of Buchan et al [15], who highlighted improved NAAT sensitivity with the use of enrichment broth. Although enrichment broth is validated for use with the HG Swift machine, it increases the TAT [16], thus, limiting the opportunity to administer IAP during labor to prevent GBS transmission to the infant. The near-patient assessment of direct swabs provided evidence of the effectiveness of the HG GBS LAMP assay in a real-world setting, increasing the generalizability of the study results to other low-resource settings. This is particularly important in this context because the feasibility of using assay in Uganda was an important outcome measure of this study [20].

There were some notable limitations within our study. The GBS colonization prevalence estimate in this study was much lower in phase 1 (11.5%) than in phase 2 (27.1%). This may reflect differences in the 2 populations tested and/or the timing of the sample collection compared to laboratory analysis in the 2 phases. In phase 1, the swab samples were collected from pregnant women at delivery and after rupture of membranes, whereas in phase 2, the samples were collected in nonpregnant women. A similar prevalence estimate of 12% in pregnant women was reported by Seale et al. from their study conducted in coastal Kenya [21]. Although 1 previous study in Uganda reported GBS rectovaginal colonization prevalence of 28.8% in pregnant women in southwestern Uganda [22], this study had microbiology facilities on site. While we made every attempt to ensure that samples remained between 2 ° and 8 °C during the transportation period, is it possible that some swab samples in our study may have been affected by the temperatures outside of the transportation cool box (which were often upwards of 30 °C); thus, the viability of GBS for culture for some of the collected swabs may have been diminished. If we assume that the culture-negative LAMP-positive samples were also representative of true GBS colonization, the overall prevalence of GBS rectovaginal colonization in pregnant women among our study population rises to 30.0%, which aligns more closely with the previous estimate in this setting and globally [23].

## CONCLUSION

The results of this study indicate promising utility of the LAMP for the detection of rectovaginal GBS colonization in resource-limited settings. The use of the HG GBS LAMP assay in such settings could be transformative for the health of infants born in LMICs. Adopting this test for the detection of GBS rectovaginal colonization in LMICs could improve the effectiveness of IAP prevention strategies, especially in countries like Uganda, where testing during the antenatal period would not be practical. The HG Swift machine and accompanying equipment is compact and can therefore be readily accommodated within most antenatal clinics and labor wards and could be used in more remote healthcare settings, where access to laboratory facilities is not available. Although we have demonstrated that the HG Swift machine is easy to use and provides rapid results, the cost-effectiveness of this intervention still needs to be assessed, as high pricing would make the implementation of this assay in LMICs prohibitive. Any such test would need to be both rapid and cheap as hospital budgets in the public sector in countries such as Uganda are commonly under considerable strain. Training is also required to run the HG GBS LAMP assay and would need to be factored into any plan to implement it widely.

## Notes

***Acknowledgments*.** The authors acknowledge the contribution of the Medical Research Council Clinical Diagnostic Laboratory Services for their assistance with bacterial culture and the staff and participants at Kawempe National Referral Hospital for taking part in the study.

The complete PROGRESS Study Author Group comprises Abdelmajid Djennad, Agnes Nyamaizi, Agnes Ssali, Alexander Amone, Amusa Wamawobe, Annettee Nakimuli, Caitlin Farley, Carol Nanyunja, Christine Najuka, Cleophas Komugisha, Dan R. Shelley, Edward A. R. Portal, Ellie Duckworth, Emilie Karafillakis, Geraldine O’Hara, Godfrey Matovu, Hannah G. Davies, Janet Seeley, Joseph Peacock, Juliet Nsimire Sendagala, Katie Cowie, Kirsty Le Doare, Konstantinos Karampatsas, Lauren Hookham, Madeleine Cochet, Margaret Sewegaba, Mary Kyohere, Maxensia Owor, Melanie Etti, Merryn Voysey, Moses Musooko, Musa Sekikubo, Owen B. Spiller, Patience Atuhaire, Paul T. Heath, Philippa Musoke, Phiona Nalubega, Pooja Ravji, Richard Katungye, Ritah Namugumya, Rosalin Parks, Rose Azuba, Sam Kipyeko, Simon Beach, Stephen Bentley, Tim Old, Tobius Mutabazi, Valerie Tusubira and Vicki Chalker.

***Author contributions*.** K.L.D. conceived the study. J.N.S., R.A., and K.L.D. contributed equally to the study design. J.N. and K.L.D. contributed to the acquisition of the study data. J.N.S. and K.L.D. analyzed the study data. J.N.S. and M.E. wrote the first version of the manuscript. J.N.S., M.E., K.L.D., and J.P. contributed equally to subsequent revisions of the manuscript. All authors reviewed the final version of the manuscript prior to submission.

***Patient Consent Statement*.** Written informed consent was obtained from all patients involved in the study. The study has been approved by the Ugandan National Council for Science and Technology (UNCST; ref. HS 2496), Makerere University School of Medicine Research & Ethics Committee (SOMREC; ref. 2018–130), and St George's University of London (SGUL REC, ref. 2020.0024), who sponsored the study.

***Financial support*.** This work was supported by the Bill and Melinda Gates Foundation [OPP1189053]. The funder had no role in the study design, data collection and analysis, decision to publish, or preparation of the manuscript.

***Supplement sponsorship***. This article appears as part of the supplement “Neonatal and Young Infant Infection in Uganda: the PROGRESS Study,” sponsored by St George's, University of London.
